# Analysis of macular structure in age-related cataract patients with different antibody levels of severe acute respiratory syndrome coronavirus-2 vaccine

**DOI:** 10.3389/fimmu.2022.1024124

**Published:** 2022-11-09

**Authors:** Xiaochun Li, Xiaoguang Cao, Zhongting Pan, Xinping Sun, Yongzhen Bao

**Affiliations:** ^1^ Department of Ophthalmology, Peking University People’s Hospital, Eye Diseases and Optometry Institute, Beijing Key Laboratory of Diagnosis and Therapy of Retinal and Choroid Diseases, College of Optometry, Peking University Health Science Center, Beijing, China; ^2^ Department of Ophthalmology, Peking University International Hospital, Beijing, China; ^3^ Clinical Laboratory, Peking University International Hospital, Beijing, China

**Keywords:** COVID-19 vaccine, antibody, macula, ETDRS zoning, retina

## Abstract

**Objective:**

To analyze the macular structure of age-related cataract (ARC) patients with different antibody levels after COVID-19 vaccine injection, in order to obtain the effect of COVID-19 vaccine on the macular structure, and speculate whether the COVID-19 vaccine has adverse effects on the macular structure.

**Methods:**

This retrospective study is conducted to analysis on the status of COVID-19 vaccine and the thickness of different layers at different positions in the macular area of ARC patients. In the age, sex and eye axial length matched population, in the un-injection, no-antibody, IgM and IgG positive groups after vaccination, the choroid, ganglion cell complex, nerve fiber layer and retinal thickness at different positions of ETDRS zoning in the macular area were discussed.

**Results:**

A total of 164 patients (164 eyes) were included in the analysis. There were 63 males and 101 females. The average age was 65.99 ± 8.43 years. There was no significant difference in age and sex among the groups (p>0.05). The average axial length of 164 eyes was 23.56 ± 1.46mm, and no significant difference between the groups (p>0.05). Non parametric test and ANOVA test for the thickness of choroid, retina, ganglion cell complex and retinal nerve fiber layer in each division of ETDRS showed no significant difference in the four groups of un-injection, no-antibody, IgM and IgG (p>0.05). There was no correlation between the antibody concentration and the thickness of macular structure (p>0.05).

**Conclusion:**

There was no significant difference in the thickness of choroid, retina, ganglion cell complex and retinal fiber layer in different macular areas after COVID-19 vaccine injection. There was no linear correlation between the thickness of choroid, retina, ganglion cell complex and retinal fiber layer and the antibody concentration produced after COVID-19 vaccine injection. It suggests that the injection of COVID-19 vaccine might have no significant effect on the macular structure of eye.

## Background

Novel coronavirus disease 2019 (COVID-19) is a global health disaster and a great challenge for mankind. The prevalence of COVID-19 has greatly impacted the global health system, disrupted the worldwide economy and caused incalculable losses (1). In the face of serious epidemic situation, in addition to avoiding contact with infectious sources as much as possible, the COVID-19 vaccination has brought new hope for the alleviation of epidemic. China is currently using five vaccine development technologies to develop and produce COVID-19 vaccines: whole virus inactivated vaccine, adenovirus vector vaccine, recombinant subunit vaccine, mRNA vaccine and attenuated influenza virus vector vaccine. Several studies have shown the effectiveness of different COVID-19 vaccines in inhibiting COVID-19. A meta-analysis by Zeng et al. evaluated the effectiveness of eleven COVID-19 vaccines against different variants of severe acute respiratory syndrome coronavirus 2 (SARS-CoV-2) (2). Another study reported that the efficacy of nine COVID-19 vaccines against symptomatic COVID-19 exceeded 50% (3).

COVID-19 is characterized by fever, fatigue and muscle soreness. As the disease progresses, dyspnea, respiratory failure, shock and multiple organ dysfunction may occur (4). Although ocular tissues are relatively less involved, some studies have reported ocular manifestations in patients with COVID-19 (5–9). Some studies found RNA of SARS-Cov-2 in the human ocular tissues (10–12). At present, conjunctivitis is the earliest ocular disorder caused by COVID-19 reported in the literature (13–15). COVID-19 conjunctivitis is similar to other viral conjunctivitis. At present, there is no case report of conjunctivitis affecting vision. However, the greater threat to vision is the vitreoretinopathy associated with COVID-19 infection, such as the manifestation of cotton wool spots and bleeding spots (16), flaming hemorrhage and ischemic lesions (cotton wool spots and retinal fan pallor) (17), retinal vein occlusion and retinal artery occlusion (18), resulting in macular lesions or change of thickness (16, 19–22).

COVID-19 vaccine is of great significance for the epidemic control, but its safety cannot be ignored. Some studies have reported the effects of COVID-19 vaccine on the eyes, mainly involving the posterior segment of the eyeball. The uvea, choroid and retinal vessels are most frequently affected. The median time of ocular pathological manifestations was 4 days after vaccine injected (23). The main manifestations are uveitis, white spot syndrome, central serous chorioretinopathy, retinal vein occlusion, acute macular neuroretinopathy and acute paracentric middle-level maculopathy. Other ocular manifestations include eyelid edema and rash, optic neuritis, oculomotor paralysis, etc (23). However, the causal relationship between the appearance of ocular pathology and the COVID-19 vaccine has not been confirmed by large sample and multi center research data. This study intends to analyze the macular structure of age-related cataract (ARC) patients with different antibody levels after COVID-19 vaccine injection, so as to obtain the effect of COVID-19 vaccine on the macular structure, and speculate whether the COVID-19 vaccine has adverse effects on the macular structure.

## Methods

### Recruitment of patients, and inclusion and exclusion criteria

This retrospective study is conducted in the Peking University International Hospital (PUIH), Beijing, China. The study is approved by the local ethical review board in accordance with the Declaration of Helsinki, and all patients provided the informed consent. In this study, ARC patients who were admitted to PUIH for ARC surgery, who were examined by optical coherence tomography (OCT) in the macula area and detected by COVID-19 antibody before surgery, were included. The vaccine is inactivated vaccine, which is inoculated according to personal wishes before they came to PUIH. According to whether the COVID-19 vaccine was injected or not and the antibody production after the COVID-19 vaccine injection, the differences in the macular structure of different groups were analyzed to obtain the effect of COVID-19 vaccine on the macular structure. Inclusion criteria: patients who were hospitalized in PUIH, planned to undergo ARC surgery, and who underwent macular OCT and novel coronavirus antibody detection before surgery. Exclusion criteria: Patients with severe lens opacity and unable to clearly display macular structure by OCT were excluded. Patients with severe lens opacity and unable to accurately measure the ocular axial length were excluded. Patients with anterior and posterior segment diseases such as uveitis, exudative senile macular degeneration, anterior macular membrane and glaucoma were excluded. To avoid the influence of abnormal macular structure, patients with pathological myopia were excluded. In order to reduce the possible influence of systemic diseases on the macular structure, patients with diabetes were excluded. Exclude patients with hepatitis B, hepatitis C and other vaccines in the past half year. Patients who could not obtain COVID-19 vaccination information were excluded.

### Clinical observations

Clinical history and routine clinical examination were performed by slit-lamp microscopy, indirect ophthalmoscopy, uncorrected (UCVA) and best-corrected visual acuity (BCVA) logMAR were tested, besides intraocular pressure (IOP). IOP was measured by noncontact tonometer (Canon TX-10/TX-F, Tokyo, Japan), slit lamp examination (Topcon SL-1E, Tokyo, Japan), and fundus examination (90 Dioptre, Volk Optical, Mentor, OH) with undilated pupil. All tests were performed in the outpatient of eye clinic.

### OCT measurement

Macular thickness is measured with DRI OCT Trion (Topcon Corp., Tokyo, Japan), and the radiation scanning mode is adopted for the macular area. Before the examination, the patient was dripped with tropicamide eye drops to dilate the pupils. OCT can display the thickness of choroid (CHO), retina, ganglion cell complex (inner plexiform layer + ganglion cell layer, GCL+, GCLp), retinal nerve fiber layer (RNFL) in layers. The image of the structural thickness of each layer in the macular region is represented by ETDRS partitions, which include the central region (1mm in diameter, marked with C), the inner ring region (3mm in diameter, marked with I), and the outer ring region (6mm in diameter, marked with O). The inner and outer circles are respectively divided into superior, inferior, nasal and temporal regions, which are marked as S, I, N and T. For example, ChoC as the thickness for the central region of choroid, ChoIT, ChoIS, ChoIN and ChoII as the thickness for the inner temporal, superior, nasal and inferior ring region of choroid, ChoOT, ChoOS, ChoON and ChoOI as the thickness for the outer temporal, superior, nasal and inferior ring region of choroid, and ChoT as the thickness for the total choroid.

### Detection of novel coronavirus antibody

The detection of SARS-Cov-2 antibody in this study uses the novel coronavirus (2019-nCoV) IgM antibody detection kit and novel coronavirus (2019-nCoV) IgG antibody detection kit provided by Maccura Biotechnology Co., Ltd. The determination method is micro magnetic particle chemi-luminescence immunoassay. The blood sample is mixed with the micro magnetic particle coated with the mouse anti human IgM/IgG monoclonal antibody. The antibody in the sample forms an immune complex with the mouse anti human antibody. After washing, the acridine ester labeled novel coronavirus antigen is added. After washing again, the substrate solution was added to generate a chemiluminescence reaction, and the luminescence signal value was measured to obtain the antibody content.

### Grouping

IgM antibody positive (IgG positive or negative) after injection of COVID-19 vaccine was defined as IgM group. IgG group was defined as IgG antibody positive (IgM negative) after injection of COVID-19 vaccine; The patients with negative antibody after injection of COVID-19 vaccine were defined as no-antibody group. Those who did not receive the COVID-19 vaccine were defined as the un-injection group.

### Statistical analysis

SPSS statistical software (SPSS version 20.0, Armonk, New York, USA) was used for statistical analysis. In this study, except for gender and eye, the data are expressed as mean ± IQR or mean ± SD. Chi square analysis was used to compare the gender data. Kolmogorov–Smirnov test and Shapiro-Wilk test were used to test the normality of the thickness data for each layer in the macular area. Nonparametric test and ANOVA were used to compare the differences between groups. P < 0.05 means that the difference is statistically significant.

## Results

### General information

A total of 190 patients and 338 eyes were included in this study, who had the cataract operation in May 1^st^ 2021 to May 31^st^ 2022. Among them, 81 were male and 109 were female. The average age was 65.45 ± 9.39 years. To avoid research errors, only the data of right eye for all patients were taken for statistical analysis.

A total of 164 patients with 164 eyes were included as the right eye. There were 63 males and 101 females. The average age was 65.99 ± 8.43 years. Among them, 39 were in the un-injection group, 14 were male and 25 were female, with an average age of 66.26 ± 7.74 years. There were 35 in the no-antibody group, 17 males and 18 females, with an average age of 66.89 ± 6.54 years. IgM group has 12 patients, 3 males and 9 females, with an average age of 67.25 ± 9.51 years; The IgG group consisted of 78 persons, 29 males and 49 females, with an average age of 65.27 ± 9.38 years. There was no significant difference in age and gender among the groups (p=0.739 and p=0.459). See **Table 1** for age and gender distribution information of patients in each group.

**Table 1:** General information for the included age-related cataract patients.

Groups	Un-injection	No-antibody	IgM	IgG	p
Included number	39	35	12	78	
Age (years)	66.26 ± 7.74	66.89 ± 6.54	67.25 ± 9.51	65.27 ± 9.38	0.739
Male (persons)	14	17	3	29	0.459
Female (persons)	25	18	9	49	

p values for ages (one-way ANOVA) and gender (Chi square analysis).

### Axial length (AL)

Previous studies have shown that AL may affect the structure of the macular area (24). Therefore, we made statistical analysis and comparison on AL of each group. The average AL of 164 eyes was 23.56 ± 1.46mm. Among them, AL of the un-injection group was 23.41 ± 1.14mm, of the no-antibody group was 23.65 ± 1.30mm, of the IgM group was 23.52 ± 2.42mm, and of the IgG group was 23.60 ± 1.60mm. There was no significant difference in AL between the groups (p=0.894).

### Analysis of macular structure

We tested the normal distribution of the macular structure data obtained by OCT. As some data are skew distribution, the nonparametric test is used for data statistical analysis. However, in order to avoid the false negative errors in statistics, one-way ANOVA was used for verification.

The choroidal thickness of the un-injection, no-antibody, IgM and IgG group in each division of the ETDRS table is shown in **Table 2**. The nonparametric test and ANOVA test of the thickness of each partition between these four groups showed no statistical significance (P > 0.05).

**Table 2:** Analysis of choroidal thickness in the age-related cataract patients with different COVID-19 antibody statues (median ± IQR, μm).

Group	Un-injection	No-antibody	IgM	IgG	P^a^	P^b^
ChoC	161.10 ± 108.12	189.07 ± 126.26	144.84 ± 79.85	182.14 ± 83.10	0.799	0.683
ChoIT	146.53 ± 117.91	173.50 ± 103.96	144.27 ± 107.03	172.16 ± 81.40	0.592	0.675
ChoIS	204.63 ± 124.36	199.72 ± 129.28	144.23 ± 112.89	175.76 ± 91.06	0.478	0.512
ChoIN	141.81 ± 131.05	182.12 ± 125.56	165.38 ± 114.64	165.47 ± 100.02	0.480	0.655
ChoII	168.44 ± 143.09	163.11 ± 130.50	135.93 ± 112.67	170.59 ± 117.31	0.544	0.540
ChoOT	158.73 ± 110.44	169.41 ± 113.04	117.95 ± 101.08	160.65 ± 76.42	0.388	0.318
ChoOS	184.38 ± 107.99	168.06 ± 109.08	145.73 ± 92.99	162.34 ± 82.69	0.523	0.788
ChoON	113.42 ± 96.13	126.43 ± 115.19	123.42 ± 109.54	124.03 ± 101.37	0.607	0.810
ChoOI	147.11 ± 101.76	141.14 ± 81.68	138.09 ± 89.75	160.66 ± 115.27	0.590	0.635
ChoT	158.03 ± 107.06	168.69 ± 109.76	136.72 ± 82.42	156.15 ± 96.16	0.754	0.723

ChoC as the thickness for the central region of choroid, ChoIT, ChoIS, ChoIN and ChoII as the thickness for the inner temporal, superior, nasal and inferior ring region of choroid, ChoOT, ChoOS, ChoON and ChoOI as the thickness for the outer temporal, superior, nasal and inferior ring region of choroid, and ChoT as the thickness for the total choroid. Unit of thickness: μm. P^a^: p > 0.05 as nonparametric test. P^b^: p > 0.05 as one-way ANOVA test.

See **Table 3** for the retinal thickness of the un-injection, no-antibody, IgM and IgG group in each division of the ETDRS table. Nonparametric test and ANOVA test showed no significant difference between these four groups. (P > 0.05). **Table 4** shows the thickness of ganglion cell complex in each division of ETDRS in the un-injection, no-antibody, IgM and IgG groups. Nonparametric test and ANOVA test showed no significant difference between these four groups. (P > 0.05). **Table 5** shows the thickness of retinal nerve fiber layer of un-injection, no-antibody, IgM and IgG groups in each division of ETDRS table. Nonparametric test and ANOVA test showed no significant difference between these four groups. (P > 0.05).

**Table 3:** Analysis of retinal thickness in the age-related cataract patients with different COVID-19 antibody statues (median ± IQR, μm).

Group	Un-injection	No-antibody	IgM	IgG	P^a^	P^b^
RetinaC	229.16 ± 28.23	218.32 ± 35.12	219.45 ± 36.53	223.56 ± 36.16	0.879	0.665
RetinaIT	285.66 ± 17.66	287.17 ± 27.43	289.19 ± 31.51	284.07 ± 28.31	0.815	0.815
RetinaIS	300.16 ± 16.51	299.21 ± 20.81	299.74 ± 30.50	299.71 ± 24.44	0.551	0.365
RetinaIN	303.16 ± 16.75	296.52 ± 25.17	298.72 ± 41.50	301.41 ± 27.13	0.635	0.191
RetinaII	296.73 ± 24.02	294.77 ± 24.28	293.54 ± 32.50	296.54 ± 27.67	0.637	0.726
RetinaOT	251.04 ± 14.86	248.96 ± 25.12	254.74 ± 25.01	247.45 ± 27.09	0.303	0.886
RetinaOS	267.76 ± 19.43	263.02 ± 24.52	257.56 ± 19.81	266.28 ± 17.90	0.470	0.522
RetinaON	283.28 ± 22.65	273.61 ± 33.27	274.37 ± 21.10	278.17 ± 23.51	0.341	0.658
RetinaOI	256.45 ± 20.23	248.92 ± 23.70	255.62 ± 18.43	253.70 ± 24.25	0.636	0.534
RetinaT	270.24 ± 16.46	264.02 ± 24.17	268.60 ± 20.61	268.23 ± 22.43	0.658	0.447

RetinaC as the thickness for the central region of retina, RetinaIT, RetinaIS, RetinaIN and RetinaII as the thickness for the inner temporal, superior, nasal and inferior ring region of retina, RetinaOT, RetinaOS, RetinaON and RetinaOI as the thickness for the outer temporal, superior, nasal and inferior ring region of retina, and RetinaT as the thickness for the total retina. Unit of thickness: μm. P^a^: p > 0.05 as nonparametric test. P^b^: p > 0.05 as one-way ANOVA test.

**Table 4:** Analysis of ganglion cell complex thickness in the age-related cataract patients with different COVID-19 antibody statues (median ± IQR, μm).

Group	Un-injection	No-antibody	IgM	IgG	P^a^	P^b^
GCLC	38.37 ± 10.47	38.00 ± 14.95	36.28 ± 14.76	38.73 ± 15.52	0.959	0.836
GCLIT	74.76 ± 14.73	78.18 ± 25.43	74.49 ± 28.57	77.72 ± 28.94	0.921	0.747
GCLIS	83.93 ± 12.98	85.79 ± 17.20	86.02 ± 13.60	86.25 ± 17.50	0.672	0.926
GCLIN	84.54 ± 15.41	85.71 ± 17.17	81.22 ± 36.07	84.67 ± 20.44	0.474	0.277
GCLII	82.54 ± 18.48	86.67 ± 17.68	79.52 ± 31.37	85.55 ± 18.52	0.887	0.797
GCLOT	67.47 ± 17.70	62.01 ± 18.30	62.60 ± 11.49	65.65 ± 23.06	0.487	0.900
GCLOS	61.86 ± 8.02	64.60 ± 9.64	63.64 ± 10.95	61.48 ± 13.04	0.651	0.962
GCLON	66.04 ± 12.79	64.93 ± 12.84	68.74 ± 20.89	62.93 ± 14.79	0.165	0.436
GCLOI	59.09 ± 11.00	57.55 ± 12.63	59.59 ± 13.75	58.69 ± 12.65	0.623	0.927
GCLT	67.38 ± 10.45	65.86 ± 16.07	66.45 ± 14.82	66.78 ± 15.89	0.600	0.847

GCL ganglion cell complex. GCLC as the thickness for the central region of GCL, GCLIT, GCLIS, GCLIN and GCLII as the thickness for the inner temporal, superior, nasal and inferior ring region of GCL, GCLOT, GCLOS, GCLON and GCLOI as the thickness for the outer temporal, superior, nasal and inferior ring region of GCL, and GCLT as the thickness for the total GCL. Unit of thickness: μm. P^a^: p > 0.05 as nonparametric test. P^b^: p > 0.05 as one-way ANOVA test.

**Table 5:** Analysis of retinal nerve fiber layer thickness in age-related cataract patients with different COVID-19 antibody statues (median ± IQR, μm).

Group	Un-injection	No-antibody	IgM	IgG	P^a^	P^b^
RNFLC	6.17 ± 6.02	5.25 ± 5.45	5.22 ± 5.69	5.04 ± 4.58	0.167	0.499
RNFLIT	21.26 ± 9.62	21.32 ± 13.97	20.85 ± 18.89	20.18 ± 14.05	0.731	0.744
RNFLIS	25.98 ± 9.11	27.82 ± 8.71	26.02 ± 5.76	26.52 ± 7.56	0.750	0.493
RNFLIN	23.50 ± 7.97	22.81 ± 7.31	22.44 ± 8.89	23.82 ± 7.66	0.692	0.476
RNFLII	27.16 ± 8.41	28.08 ± 10.20	24.03 ± 9.97	26.34 ± 7.18	0.329	0.752
RNFLOT	23.61 ± 6.82	24.58 ± 13.41	26.96 ± 15.99	23.10 ± 12.10	0.659	0.666
RNFLOS	41.45 ± 14.13	39.76 ± 13.92	37.20 ± 11.64	40.66 ± 11.20	0.338	0.778
RNFLON	49.53 ± 15.83	50.85 ± 16.02	44.80 ± 13.88	49.24 ± 15.38	0.652	0.667
RNFLOI	42.01 ± 11.64	40.73 ± 14.07	39.74 ± 16.55	41.12 ± 10.29	0.452	0.796
RNFLT	36.01 ± 9.07	36.71 ± 9.77	35.87 ± 10.79	35.39 ± 8.77	0.426	0.442

RNFL retinal nerve fiber layer. RNFLC as the thickness for the central region of RNFL, RNFLIT, RNFLIS, RNFLIN and RNFLII as the thickness for the inner temporal, superior, nasal and inferior ring region of RNFL, RNFLOT, RNFLOS, RNFLON and RNFLOI as the thickness for the outer temporal, superior, nasal and inferior ring region of RNFL, and RNFLT as the thickness for the total RNFL. Unit of thickness: μm. P^a^: p > 0.05 as nonparametric test. P^b^: p > 0.05 as one-way ANOVA test.

The correlation analysis between the antibody concentration after COVID-19 vaccine injection and the thickness of each layer in the macular region was performed on 90 patients (90 eyes) in the antibody group (IgM group + IgG group), 12 patients (12 eyes) in IgM group, and 78 patients (78 eyes) in the IgG group. The results showed that there was no correlation between the antibody concentration of IgM group, IgG group or antibody group and the thickness of each layer for the macular structure (P > 0.05).

## Discussion

In 2019, novel coronavirus pneumonia (COVID-19) began to rage. With the variation of sars-cov-2 virus strain, the severity of the disease and the immune escape ability of the virus are also constantly changing. In the face of the serious epidemic situation, in addition to avoiding contact with infectious sources as much as possible, COVID-19 vaccination has brought new hope for the alleviation of the epidemic situation (2, 25). At present, China is using five vaccine development technologies to develop and produce COVID-19 vaccine: whole virus inactivated vaccine, adenovirus vector vaccine, recombinant subunit vaccine, mRNA vaccine and attenuated influenza virus vector vaccine.

Inactivated vaccines are most widely used in China, accounting for more than 85%. Among them, two vaccine manufacturers, BBIBP-CorV and CoronaVac, are the most widely used vaccines (26). A retrospective cohort study showed that the resistance rate to infection was 51%, the resistance rate to COVID-19 pneumonia was 61%, and the resistance rate to severe COVID-19 was 82% (26). Completing two doses of inactivated vaccine can reduce the risk of COVID-19 from mild to moderate to severe by 74%. The efficacy of BBIBP-CorV and CoronaVac vaccines was similar. 164 right eye patients were included in our study, of which 39 had no history of vaccine injection, and 125 had been injected with COVID-19 vaccine, with an injection rate of 76.2%. Although the samples are only from age-related cataract diseases in one hospital in Beijing, the proportion of vaccine injection in the samples also indicates that the current vaccine injection in China has reached a certain proportion, which provides a certain basis for epidemic control.

IgM is mainly produced in the primary immune response to infectious factors or antigens, as IgG mainly produced in the secondary. Studies have shown that SARS specific antibodies are produced in the second week of infection and last for a long time, while IgM is transiently expression (27). IgM is an antibody in the acute phase, which generally appears 3-5 days after the onset of the disease, remains positive for about one month, and then gradually decreases. IgG is a kind of recovery antibody and the main component of neutralizing antibody. The detection time of IgG is generally several days later than that of IgM, and it reaches the peak several weeks after the onset, and it can last for months or even years (28). Some studies analyzed the coronavirus antibody in the serum of patients with COVID-19. It was found that IgM antibody and IgG antibody appeared one after another in the first to second weeks after sars-cov-2 infection. After 3 weeks, IgM antibody of some individuals gradually disappeared and IgG antibody continued to exist (28). The kit of gold immunochromatography say (GICA) (Innovita Biotechnology Co., Ltd. Tangshan, China) was used in this study.

Among the 164 patients included in our study, 125 had a previous history of COVID-19 vaccine injection, including 12 (9.6%) patients with IgM antibody positive, 78 (62.4%) patients with IgG antibody positive, and 35 (28%) patients with negative antibody test. The total antibody positive rate was 72%, suggesting that COVID-19 vaccine can induce the immune response against sars-cov-2 virus antigen *in vivo*. However, it is worth noting that the antibodies measured by the clinical antibody kit are not neutralizing antibodies to sars-cov-2. Therefore, the negative antibody does not mean that the body has no immunity to sars-cov-2. Currently, inactivated vaccines are the main COVID-19 vaccines in China, accounting for more than 85%. The inactivated vaccine might need to be injected with booster injections before it can produce sufficient immune effect. It is speculated that some of the vaccine antibody negative patients may be related to not receiving intensive injection therapy. However, due to the age of the included patients, it is difficult to collect information such as vaccine injection time and injections. This study did not obtain enough information to verify this hypothesis.

Although COVID-19 vaccine injection is of great significance for epidemic control, its safety cannot be ignored. According to the report of the US vaccine adverse event reporting system, after 1893360 doses of bnt162b2 vaccine, the risk of allergic reaction was 11.1 cases/million doses, of which 71% occurred within 15 minutes after vaccination (29). The European Drug Administration recommends that the signs and symptoms of thrombosis and thrombocytopenia caused by the vaccine should be vigilant, such as shortness of breath, chest pain, leg swelling, persistent abdominal pain, neurological symptoms and minor bleeding spots under the skin outside the injection site (25).

Some studies have reported the effects of COVID-19 vaccine on the eyes. Although most of the studies are from case reports, the causal relationship between adverse reactions and COVID-19 vaccine injection events has not been determined. However, with the widespread popularity of vaccine injection, it is important to understand the effects of the vaccine on the eyes (30). At present, the ocular manifestations after COVID-19 vaccine injection mainly involve the posterior segment of the eyeball. The uvea, choroid and retinal vessels are most frequently affected. The median time of ocular adverse reactions was 4 days after the vaccine injection, and the main manifestations were uveitis, white spot syndrome, central serous chorioretinopathy, retinal vein occlusion, acute macular Neuroretinopathy and acute paracentric middle maculopathy. Other ocular manifestations include eyelid edema and rash, optic neuritis, oculomotor paralysis, etc (23). It is speculated that the possible pathogenesis includes molecular simulation of vaccine components and host ocular tissues, antigen-specific cells and antibody mediated hypersensitivity to viral antigens and adjuvants present in the vaccine (23). The causal relationship between ocular signs and symptoms and COVID-19 vaccine has not been determined, and long-term observation and analysis of large sample multicenter data are still needed.

Our study found that after matching gender, age, and axial length, there was no statistical difference in the thickness of each layer in macular structure between vaccinated and unvaccinated patients. Whether the vaccine produces antibodies or not seems to have no effect on the structure and thickness of each layer in the macula. There was no significant difference between IgM group (short time after vaccine injection) and IgG group (long time after vaccine injection) in the thickness of macular structure. No inter group differences were observed even in the choroid, which was most susceptible to immune responses. Previous studies reported that the ocular complications caused by COVID-19 vaccine mostly occurred in the short term after the vaccine injection, with a median time of 4 days. However, our study found that the thickness of each layer in the macular region of IgM positive patients did not change. In antibody positive patients, there was no correlation between the concentration of antibody and the thickness of macular structure. It is suggested that the antibody production induced by the immune response of COVID-19 vaccine could not cause changes in the thickness of the macular layer structure. However, our study could not complete the observation of macular structure changes at the cellular and molecular level, and not analyze whether the macular supermicro structure has no change after COVID-19 vaccine injection. This is the limitation of this study, and we hope that pathological studies can further confirm it.

Most of the previous reports about the effects of COVID-19 vaccine on the eyes were case reports. Our study first analyzed the effects of COVID-19 vaccine on the macular structure from the perspective of different antibody statues after COVID-19 vaccine injection in age-related cataract patients. But our study also has some limitations. First of all, our enrolled patients were all age-related cataract patients and were older, so there was a certain selection bias. Secondly, our research has not been able to analyze the changes of macular structure from the cytological level. We hope that there will be more advanced research in the future to achieve this goal. Thirdly, our sample size is still small, and domestic COVID-19 vaccines are mainly inactivated vaccines, which cannot represent the effects of all COVID-19 vaccines. Therefore, further and more advanced research is needed.

## Data availability statement

The original contributions presented in the study are included in the article/supplementary material. Further inquiries can be directed to the corresponding author.

## Ethics statement

The studies involving human participants were reviewed and approved by Peking University International Hospital. The patients/participants provided their written informed consent to participate in this study.

## Author contributions

XL and XC wrote the main manuscript text, and prepared figures and tables. ZP and XS provided the part of data. YB supervised the writing. All authors reviewed the manuscript.

## Funding

This study was supported by the National Natural Science Foundation of China (NSFC, No. 21173012) and National Key R&D Program of China, No.2020YFC2008200.

## Conflict of interest

The authors declare that the research was conducted in the absence of any commercial or financial relationships that could be construed as a potential conflict of interest.

## Publisher’s note

All claims expressed in this article are solely those of the authors and do not necessarily represent those of their affiliated organizations, or those of the publisher, the editors and the reviewers. Any product that may be evaluated in this article, or claim that may be made by its manufacturer, is not guaranteed or endorsed by the publisher.
